# An Experimental Investigation of the Material Properties of the A356 Aluminum Alloy Power Fittings in the Vacuum Die-Casting Process

**DOI:** 10.3390/ma17061242

**Published:** 2024-03-08

**Authors:** Jianli Zhao, Yilin Wang, Xiaowei Wang, Yisheng Zhang

**Affiliations:** 1 Inner Mongolia Power Research Institute Branch, Inner Mongolia Power (Group) Co., Ltd., Hohhot 010020, China; jianli791026@126.com; 2 Key Laboratory of Material Forming and Mold Technology, Huazhong University of Science and Technology, Wuhan 430074, China; m202371039@hust.edu.cn (X.W.); zhangys@hust.edu.cn (Y.Z.)

**Keywords:** power transmission, transformation fitting, A356 aluminum alloy, vacuum die-casting process (VDCP), process optimization

## Abstract

To enhance the performance of ultra-high voltage power fittings in severe weather conditions without altering their current structure, the high-strength and toughness aluminum alloys were rationally selected to study the optimization of the die-casting process. This approach aims to improve the overall longevity and function of the power fittings in extreme climates. First of all, the propose of this study is to use the material’s strength–toughness product (STP) concept to evaluate the material stability of the power fitting impact resistance and fatigue toughness in order to determine the appropriate material selection. Secondly, the location of the mold’s sprue and gate was optimized through finite element simulation to prevent gas volume and flow defects during the casting process. This improves the material’s toughness and anti-fatigue failure characteristics of the product. Then, vacuum equipment and a vacuum valve auxiliary system were added based on the existing die-casting machine, and the mold structure was optimized to enable the vacuum die-casting process. Finally, a water-based boron nitride environmentally friendly mold release agent was used to solve demolding difficulties with an A356 aluminum alloy and improve mold lubrication and surface quality. The production of quad-bundled spacers using A356 and vacuum die casting has resulted in parts with a tensile strength of at least 250 MPa and an elongation of no less than 7%. This improvement has laid a foundation for enhancing the operational reliability of existing overhead transmission line fittings.

## 1. Introduction

Power fittings refer to the metal accessories used in the mechanical connection, fixing, and protection of overhead transmission and transformation lines. There are many types of power fittings, mainly including connection fittings, tension wire clamps, overhanging wire clamps, and protection fittings [1]. Power fittings are crucial for power transmission and transformation projects, as their reliability and stability directly impact the safety of the power grid system. In recent years, China’s ultra-high voltage transmission network construction has continuously expanded. Consequently, the transmission lines traverse areas of different terrain, making them increasingly susceptible to complex and changeable meteorological conditions. As a result, the power fittings demand higher performance, mechanical properties, reliability, durability, energy efficiency, and cost effectiveness. The material selection of power fittings is one of the most critical factors in determining their comprehensive performance.

Currently, power fittings are commonly made from malleable cast iron, steel, aluminum, and aluminum alloys. Malleable cast iron and steel hold a dominant position due to their favorable mechanical properties and economical cost. The State Grid Corporation of China mandates cast iron fitting materials to exhibit a tensile strength exceeding 330 MPa. In reality, the cast iron and steel fittings utilized in the power industry have a tensile strength greater than 360 MPa. This includes Q234A, 35 steel, and 40 steel, with tensile strengths of 370 MPa, 530 MPa, and 570 MPa, respectively. These strengths surpass the standard’s specified strength requirements.

However, cast iron fittings have revealed a series of challenges in daily operation and maintenance. Firstly, iron fittings are frequently thick and bulky, causing significant inconvenience in transportation and installation, resulting in increased costs. Secondly, cast iron fittings have poor corrosion resistance, often requiring the application of hot-dip galvanizing to enhance their corrosion resistance. However, the hot-dip galvanizing process produces liquid waste that can severely pollute the environment. Furthermore, the galvanized layer is susceptible to detachment from external mechanical forces, consequently exposing the cast iron matrix and significantly diminishing the fixture’s corrosion resistance [2,3]. Thirdly, cast iron fittings exhibit significant hysteresis and eddy current losses, resulting in substantial reductions in power utilization efficiency [4]. As ferromagnetic materials, cast iron fittings form a closed loop around the wire. Passing alternating current through a wire generates an alternating magnetic field around it, which induces an eddy current inside the fixture, resulting in a power loss known as eddy current loss. According to research conducted by the Ohio Brass Company in the U.S., ferromagnetic fittings cause power loss at a rate of about 0.01% to 0.03% of the transmission capacity. Ferromagnetic power connectors result in significant power loss, leading to substantial energy and economic waste. The increased temperature within the connectors due to this loss reduces their mechanical strength and long-term safety, reliability, and durability. Therefore, the development of power fittings that are energy efficient is essential.

Compared to iron fittings, aluminum fittings offer several advantages [5,6]. Firstly, aluminum has a density that is only one-third of that of iron, reducing the weight of the fittings and making them easier to transport and maintain. Additionally, this density reduction results in a lower line load. Secondly, the aluminum fittings offer excellent resistance to corrosion. During service, they can easily form a thick layer of alumina passivation film on the surface, which effectively blocks the diffusion of oxygen and corrosive elements to the underlying substrate, thereby reducing the risk of further oxidation corrosion.

An aluminum alloy, a non-magnetic material, effectively reduces hysteresis and eddy current losses in fittings during use. In their analysis using the finite element simulation method, Cheng et al. [7] compared the magnetic flux density, eddy current density, and energy consumption of cast iron and aluminum alloy fittings. The result indicates that the energy consumption of aluminum fittings is noticeably lower than that of cast iron fittings, with only 5–10% of their energy consumption. Li et al. [8] employed finite element simulation to determine the power consumption of wire clips made of cast iron and an aluminum alloy when exposed to various levels of energizing currents. The findings indicate that the energy consumption of aluminum alloy clips was significantly lower than that of cast iron clips when subjected to the same current. Further analysis suggests that the implementation of aluminum alloys for suspension clips instead of cast iron can reduce energy consumption, resulting in savings in electricity costs. In fact, the cost savings can fully compensate for the increased cost of aluminum alloys within fewer than 12 years. Currently, aluminum fittings are being widely utilized in power systems both domestically and internationally. China’s 500 kV ultra-high voltage transmission lines have frequently implemented aluminum wire clamps. Countries such as Russia and the United States make extensive use of aluminum fittings on their high-voltage transmission lines.

However, there are many problems with aluminum fittings. (1) Typically, the cost is three to five times greater than that of malleable cast iron and cast steel fittings. (2) The low mechanical strength of aluminum fittings makes it challenging to meet the electric power industry’s demands. Existing common aluminum alloys, such as commonly used ZL102, ZL101A, etc., have a tensile strength of only 140 MPa or so, significantly lower than the State Grid Corporation for malleable cast iron fitting strength requirements.

Wear is the prevailing cause of power fittings’ failure [9,10]. Li and colleagues [11] conducted a study on the wear and tear of insulator umbrella skirts, U-shaped rings, and other fittings in 750 kV transmission lines. The study involved combining actual fault data to analyze wear patterns in these components. The findings demonstrate that strong winds and dust elevate the pressure on the connecting fittings, causing even minor surface defects to result in fractures. Additionally, the twisting of rings due to strong winds further contributes to wear and tear and increases the likelihood of failure of the connecting fittings, insulators, and other power fittings. In response to the increasing number of accidents caused by fitting failure in large temperature difference environments, Wu et al. [12] conducted a study on the causes of frost cracking and preventive measures for electric power fittings. They discovered that the theoretical strain on fitting design is smaller than that of fittings in the iced state. Additionally, they found that fitting fractures occur under the influence of alternating heat and cold. To enhance the anti-wear performance of the fitting, it is advisable to use wear-resistant materials with high tensile strength or to modify the surface of the fitting to extend its service life.

Existing casting material toughness is low; if the die-casting process control is not good, the aluminum alloy fittings will not meet the long-term use requirements [13,14]. Extreme weather is a prominent cause of power system accidents, as it can lead to significant damage. One contributing factor to such damage is the lack of sufficient low-temperature toughness in power fitting components, rendering them unable to withstand the impacts of extreme weather changes.

**Figure 1:**
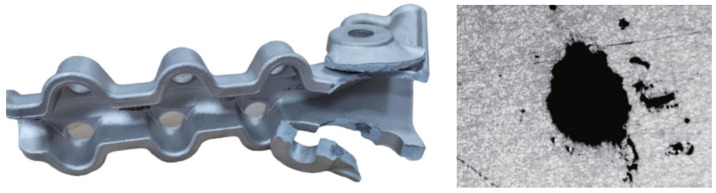
Tension clamp fracture and porosity in the cast structure.

Developing high strength and toughness in aluminum alloy materials is essential for the production of high-performance and lightweight fittings. Additionally, the utilization of advanced forming process technology is crucial for their development. Existing power fittings typically employ either a low-pressure-casting or high-pressure-casting process. To enhance product quality and minimize defects, it is necessary to incorporate slagging and degassing processes during aluminum alloy refining to reduce gas and slag levels. Because of the high production efficiency and dimensional accuracy, high-pressure die-casting has become a commonly used forming process in power-fitting manufacturing. Die casting often led to porosity, shrinkage, cold segregation, and wrong flow as the main casting defects. Apart from shrinkage, all other defects are related to the air trapped in the feeding process [15]. Figure 1 displays a typical tension clamp fracture during a tension test and illustrates the microstructure of the faults.

Because of gas problems, ordinary die castings result in elongation not being high enough; therefore, high-vacuum die-casting (HVDC) technology has received wide attention [16,17,18]. HVDC offers benefits, such as decreased porosity, improved surface quality, and higher elongation rates, while maintaining the same production efficiencies as conventional die casting. Moreover, it can enhance eutectic silicon morphology and generate precipitation reinforcement through heat treatment, thereby further improving toughness. The utilization of HVDC can significantly enhance casting quality and streamline casting processes, and thus is widely employed in the automotive parts manufacturing sector. As vacuum die-casting equipment and technology continue to advance, their application areas are expected to expand further.

The optimal approach to enhancing the die-casting process involves upgrading the vacuum pressure equipment within the existing die-casting machine system. This can be achieved without modifying the original production line configuration, resulting in improved product quality and reliability. Second, the use of die-casting simulation software allows for the simulation of the entire injection cycle and the prediction of the flow path and defects. This helps optimize the sprues or runners of the mold and gas riser position to reduce molding defects and improve the part’s tensile strength and overall productivity.

The A356 aluminum alloy is a sub-eutectic-casting aluminum alloy that contains reinforcing elements of Si and Mg in the Al-Si system. Due to its exceptional qualities, such as low density, excellent formability, and corrosion resistance, this alloy finds applications in various fields, such as automotive, aerospace, spacecraft manufacturing, etc. [19,20,21]. However, there is no existing literature on the utilization of this alloy in the production of power fittings. This paper proposes utilizing the A356 aluminum alloy combined with the VDCP to create power fittings with improved strength and toughness.

Later aging treatment can improve the mechanical properties of certain die-casting aluminum alloys [22]. According to [23], the tensile and yield strength of AlSiMgMn alloy parts produced through high-vacuum die casting increased after T6 heat treatment. However, during the heat treatment process, the casting may experience some minor deformations. In the case of large and complex die casting, these deformations may be further amplified, resulting in a final product that does not meet the design requirements. Therefore, aging treatment will not be used in this experimental study.

The enhancement of material strength can lead to a reduction in toughness. Some components with high strength may not exhibit better fatigue performance during service. Choosing the optimal material with a balanced toughness–strength ratio is crucial for ensuring good safety performance during service. This has become a challenging problem in engineering. Since the strength and elongation of the material can be easily obtained through a room temperature tensile test, the strength and toughness product (tensile strength and elongation of the product) can be used as a comprehensive index to express the toughness of the material. Evaluating the applicability of the material and its forming process with a strength–toughness product (STP) can effectively guide the selection of the material and forming process.

## 2. Materials and Methods

### 2.1. Material Specifications

ZL102 is a conventional cast material with a typical composition, as shown in Table 1. After die casting, the ZL102 aluminum alloy exhibits mechanical properties with a tensile strength (Rm) greater than 155 MPa and an elongation of at least 2%. In this study, ZL102 will be used as the reference material for comparison.

Considering the aforementioned factors, it is proposed to use the A356 aluminum alloy instead of the ZL102 aluminum alloy. The A356.2 aluminum alloy (composition shown in Table 2) has a lower copper content (not greater than 0.10%) compared to the ZL102 aluminum alloy (not greater than 0.30%), which helps to improve the atmospheric corrosion resistance of the fittings. The toughness of materials can be improved through the slagging and degassing process and the VDCP method. This method will overcome gas and slag defects and result in casting parts with an Rm greater than 195 MPa and an elongation greater than 5%.

### 2.2. Process Design and Simulation

**Figure 2:**
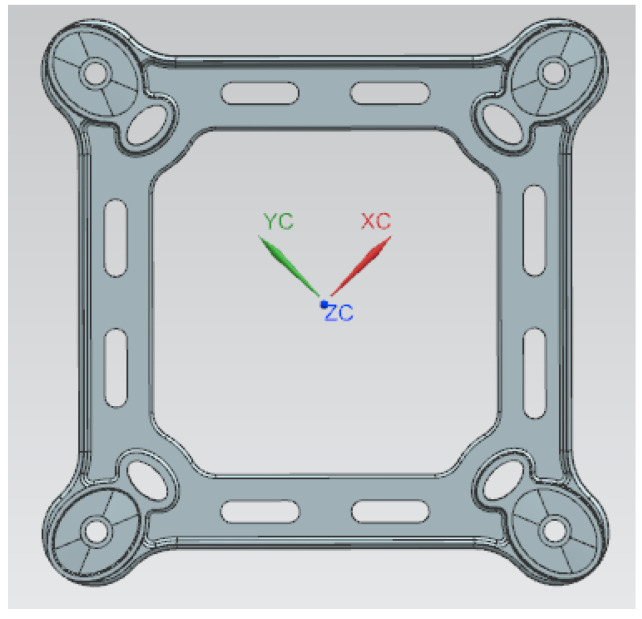
Three-dimensional modeling of the main frame component.

**Figure 3:**
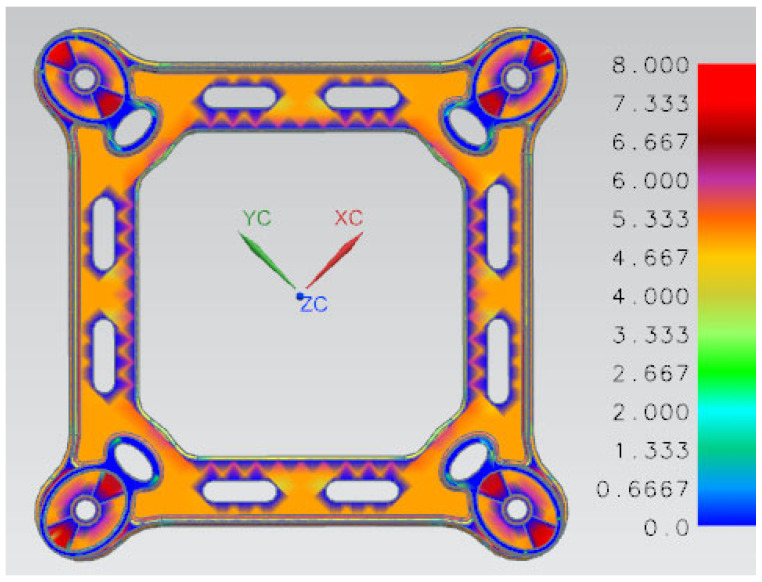
Thickness distribution map of the main frame component.

A spacer bar is a protective device used in transmission lines to maintain the relative position of the sub-conductors. It prevents the sub-conductors from whipping each other by keeping a certain distance between the split sub-conductor bundles. Additionally, the spacer bar helps to suppress transmission line breeze vibration. In the event of a line short-circuit fault, the spacer bar should not be permanently deformed. After the short-circuit current disappears, the spacer bar should be able to restore the sub-conductor design spacing. The spacer bar must have a split sub-conductor wire clamp with sufficient grip to prevent movement in the direction of the line during long-term operation. It is important to consider comprehensive performance indicators, such as line short-circuit split sub-conductor centripetal force, wind load transverse tensile pressure, and long-term wind vibration fatigue damage of the wire. The quad-bundled spacer is a commonly used type. Figure 2 shows the main frame component of this type of spacer. After analyzing the wall thickness, it was found that the majority of the area has a thickness of 5 mm, with the thickest point being connected to the jaws at a thickness of 8 mm, as shown in Figure 3.

**Figure 4:**
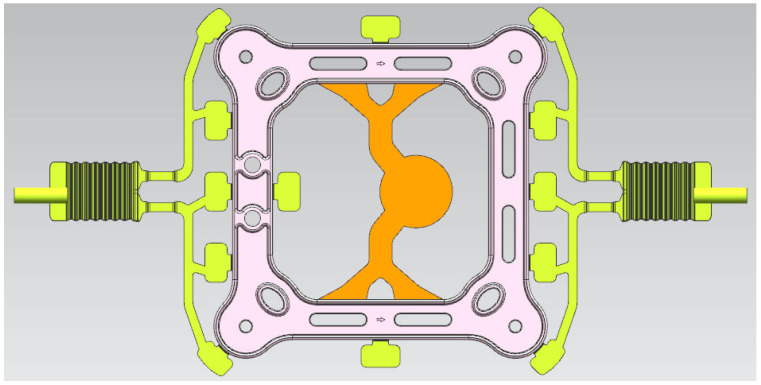
The overall structure of the part, the casting system and the overflow system.

The overall structure of the casting system and the overflow system is shown in Figure 4. The quad-bundled spacer’s structural symmetry necessitates the design of two main cross-gates. These gates have different cross-sectional areas to control the flow rate and ensure smooth filling of the liquid metal in the cavity. To achieve this, a fan-shaped sprue is used, and the inner gate is located at the combination of the horizontal runner and the casting. The overflow channel is located at the end of the liquid metal filling. Compared to ordinary die casting, vacuum die casting also requires consideration of the position of the vacuum valve. In the past decade, the mainstream method for exhausting air in a pressure chamber has been to connect the vacuum valve directly to the exhaust groove and add a pumping cut-off valve, known as the labyrinth device, in between.

The casting molding simulation method based on the proCAST finite element is as follows:

① Generate an STL model file through 3D CAD modeling;
② Import the STL model file into proCAST v.17.5.0 software and generate a mesh model with a minimum mesh size of 1.00 mm;
③ Set the material parameters for material A356, including an alloy density of 2500 kg/m^3^;
④ Set process parameters, including an initial temperature of 650 °C and a solidification time of 15 s;
⑤ Finally, perform the computational simulation.

To avoid casting shrinkage defects, the optimal location of the gate, the number of gates, and the best process parameters for die-casting production should be selected based on repeated calculation simulations, analyses, and comparisons of different process parameters.

### 2.3. Trial Production and Performance Testing

**Figure 5:**
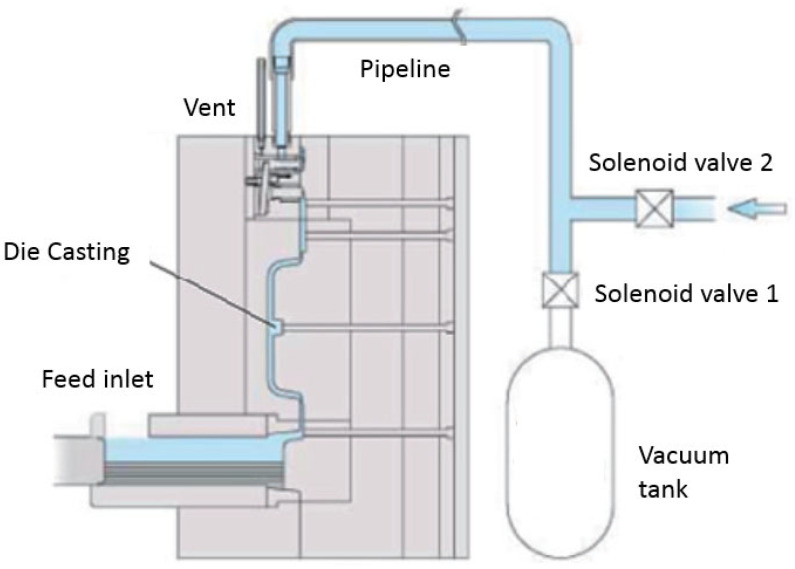
Vacuum die-casting machine.

We added the vacuum equipment and vacuum valve auxiliary system to the existing 300 T die-casting machine, as shown in Figure 5. The vacuum tank should be operated at an air pressure of 20~27 kPa. When solenoid valve 1 is activated, the exhaust end of the mold is connected to the vacuum tank, and the air pressure in the mold is reduced to a level close to the vacuum tank pressure within 0.3~0.5 s. This operation helps to maintain optimal internal conditions of the mold cavity for die casting. The initial die-casting temperature is set to approximately 650 °C, with a die-casting cycle time of 13 s. In engineering applications, a set of vacuum generators and vacuum tank equipment can be connected to multiple existing die-casting machines through pipelines and control valves to improve equipment utilization and reduce costs.

**Figure 6:**
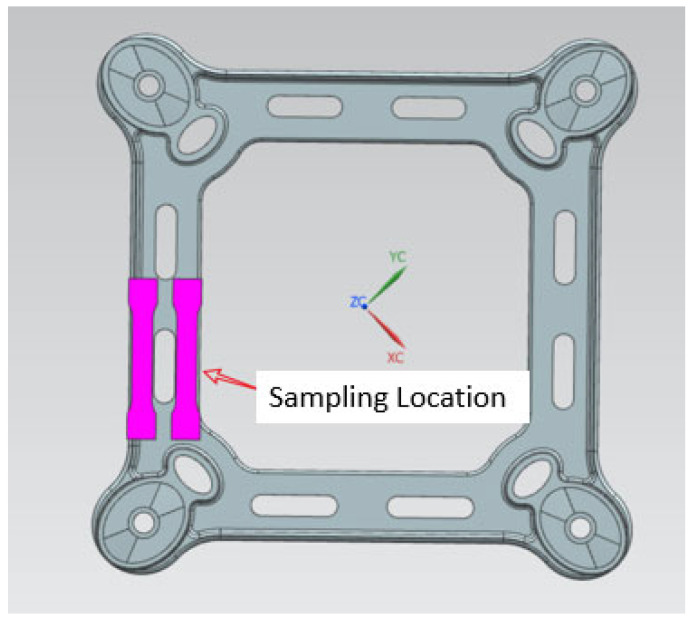
Location of spacer bars for taking tensile samples.

**Figure 7:**
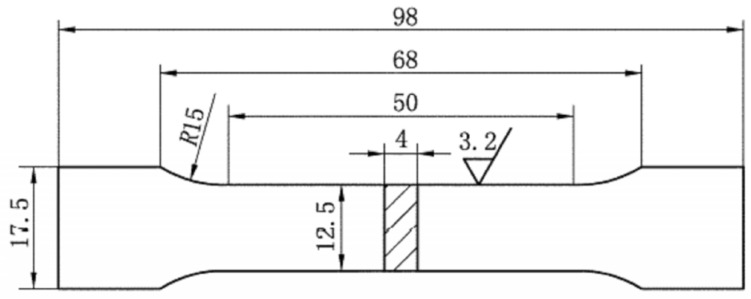
Dimensions of the tensile specimen (unit: mm).

This study aims to test the mechanical properties of spacer bars processed by different methods and materials. The three different spacer bar parts tested were (1) ZL102 processed by ordinary die casting, (2) ZL102 processed by the VDCP, and (3) A356 processed by the VDCP. The goal is to identify the materials and processes that result in spacer bars with optimal mechanical properties. The mechanical properties of the tensile specimen taken from the spacer bar were tested after it was cut in a suitable position. The location of the tensile specimen on the spacer bar is shown in Figure 6. The spacer bar clamping position is partially missing due to its size limitation. The dimensions of the tensile specimen are shown in Figure 7.

All specimens taken from aluminum alloy frame parts were tested on an AG-IC precision tensile tester at a tensile speed of 2 mm/min. Since the surface of the taken specimens was very rough to prevent the rough surface of the specimens from affecting the experimental results, they were sanded using 280 grit, 500 grit, and 800 grit sandpaper until smooth.

Based on the damaged VDCP specimen, a 10 × 10 mm sample was cut near the fracture of the specimen and analyzed for fracture morphology and composition using a thermal field emission scanning electron microscope (model: JEOL JSM-7600F, Shanghai, China). Through fracture morphology analysis, indicate if there are defects, such as porosity and inclusions in the specimen, and make suggestions to optimize specimen performance.

## 3. Results and Discussion

### 3.1. Optimizing the Casting System through Finite Element Simulation

The injection system’s optimized design is verified by presetting several gate position parameters. Changing the parameters of the main gate improves its throttling characteristics, thus altering the balance of flow velocity in each branch. This ensures the balance of the flow of aluminum liquid in the cavity, improving the cooling uniformity of the castings and avoiding shrinkage defects in the original position.

**Figure 8:**
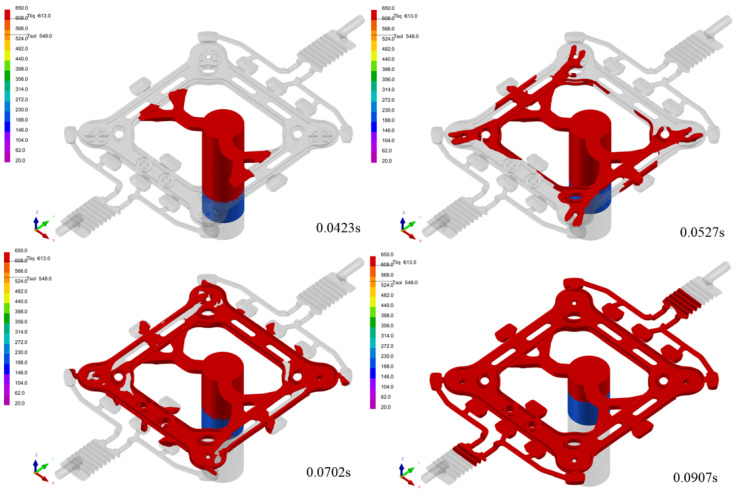
The simulation of the filling process at an injection speed of 2.5 m/s.

Numerical simulation was conducted on aluminum alloy A356 parts for vacuum die casting under the following conditions: casting temperature of 650 °C, mold initial temperature of 200 °C, and injection speeds of 1.0, 2.5, and 5.0 m/s. This study aimed to analyze the effects of these variables on the casting process. The filling time under the three kinds of injection speed is 0.2267 s, 0.0907 s, and 0.0454 s, respectively. The simulation of the filling process at the injection speed of 2.5 m/s is shown in Figure 8. When t = 0.0423 s, the liquid metal uniformly flows into the thin wall of the spacer’s frame through the inner gate, and at this time, the liquid metal is simultaneously injected from the inner edges. It illustrates the reasonableness of the design of the overall feeding of the spacer bar. When t = 0.0527 s, the liquid metal is filled from the thin wall of both sides of the part through the ring channel, and the filling progress of the left and right ends of the frame is uniform, which shows the reasonableness of setting the different cross-sectional area to control the flow of liquid metal. After t = 0.0702 s, the liquid metal finished filling the final position of the cavity.

**Figure 9:**
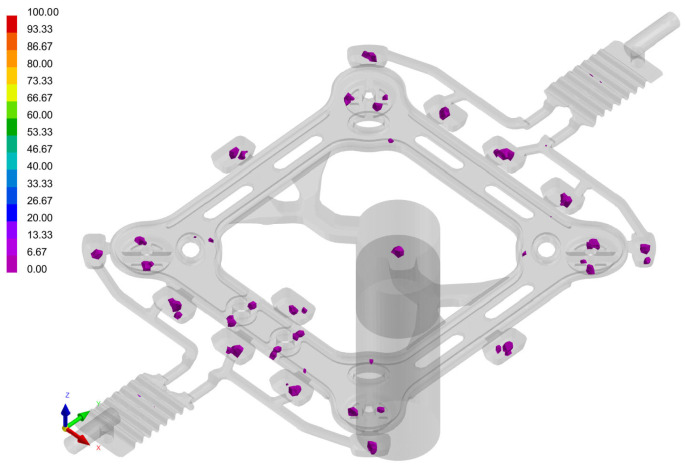
Predicted porosity distribution from the simulation at an injection speed of 2.5 m/s.

Figure 9 shows the distribution of porosity after solidification. Most of the voids are distributed in the runners and exhaust channels, and there are a few thicker areas in the part, which is basically consistent with the actual production situation.

### 3.2. Control of the Vacuum Die-Casting Process

**Figure 10:**
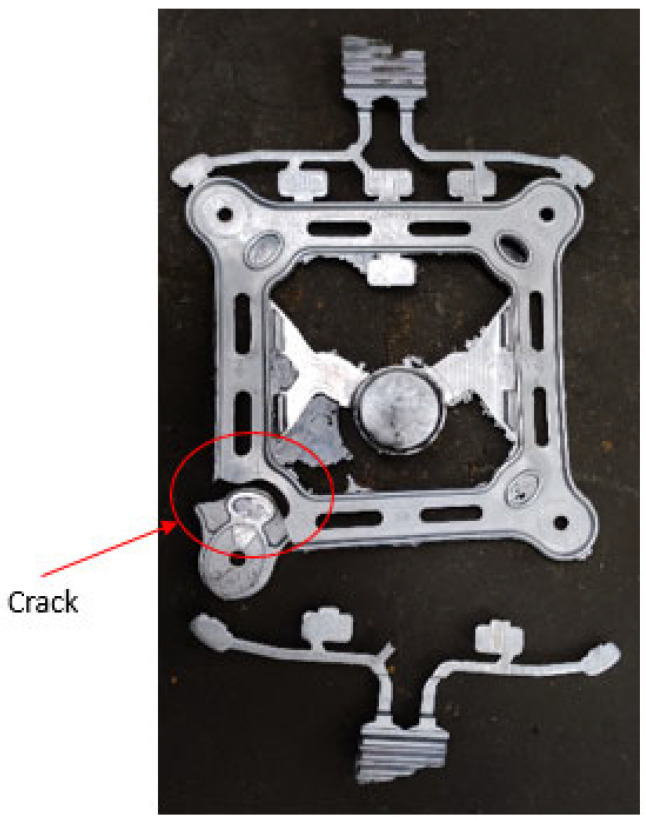
A cracked part.

The VDCP for the A356 aluminum alloy can result in parts sticking to the mold, making mold release difficult. As shown in Figure 10, severe mold sticking occurred as soon as the A356 spacer frame was produced to the third piece, and when the part was removed, part of the part stuck to the mold, and the part cracked right through.

To address this issue, it is essential to spray a mold release agent during production. Spraying can create an isolation layer on the mold surface, preventing direct adhesion between the alloy liquid and mold. This effectively reduces the scouring of the metal liquid on the mold and improves the surface quality of the die casting, resulting in a smoother casting surface. However, spraying at mold temperatures higher than 300 °C can cause the mold release agent to completely separate from the mold surface, rendering the spraying ineffective. Tests have shown that maintaining a mold temperature between 100 and 15 °C prevents an increase in cavity gas, especially when using a water-soluble diluent that can quickly evaporate. To aid in the release of A356 aluminum alloy die-casting molds, Quaker Chemical DIE SLICK @ 4514 SEW, a new water-based lubricant release agent, can be used to facilitate easy part release and reduce mold wear. The use of an environmentally friendly lubricant mold release agent can improve the surface quality of castings and significantly reduce part deformation and breakage rates. Additionally, it can enhance the dimensional accuracy and stability of mass-produced products.

To enhance the quality control standards of refining, degassing, and dross removal processes, the VDCP can be utilized to minimize the risk of forming holes or looseness due to trapped gas during die casting. Additionally, optimizing the mold structure design can reduce the formation of hot spots and resulting solidification defects. Through these improvements, the new die-casting process has significantly improved compared to the conventional die-casting process. The ratio of product defects has decreased from 1% to 0.15%.

### 3.3. Influence of the Vacuum Die-Casting Process on the Mechanical Properties of a Die-Cast Aluminum Alloy

Samples of ZL102 spacer bars were produced using an ordinary die-casting process for the purpose of conducting tensile tests. The material exhibited an average tensile strength of 145.42 MPa and an average elongation of 1.75%. The material’s performance falls short of the required tensile strength and elongation due to the presence of numerous macropores within the parts. The existence of these pores significantly reduces the material’s expected value. The ZL102 spacer bar was produced using the VDCP, and its material has an average tensile strength of 157.67 MPa and an average elongation of 3.5%. The comparison shows that there is not much increase in tensile strength, but there is a significant increase in elongation. Although a small number of air holes still exist in the material, their quantity has been greatly reduced compared to parts produced through the ordinary die-casting process.

**Figure 11:**
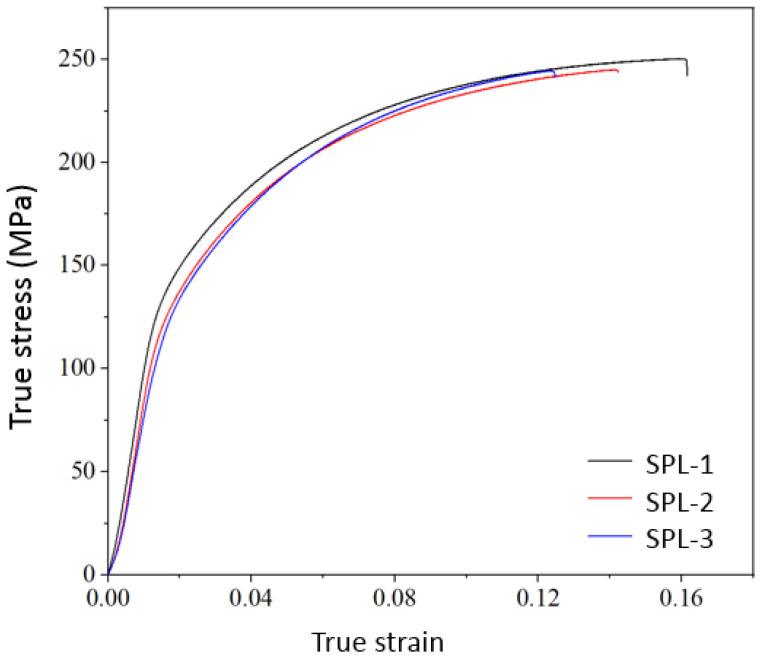
The true stress–strain tensile curve of the A356 specimens.

Tensile specimens were obtained from vacuum die-cast A356 spacer bars to test their mechanical properties. The stress–strain tensile curve obtained from the experiment is shown in Figure 11, with an average tensile strength of 246.52 MPa and an average elongation at break of 10.18%.

Table 3 compares the results of two materials with the VDCP. It is evident that the VDCP using A356 significantly enhances the comprehensive performance of power fittings. The use of advanced forming technology and high-strength–toughness aluminum alloy materials can address the current issue of insufficient toughness in power fittings.

**Figure 12:**
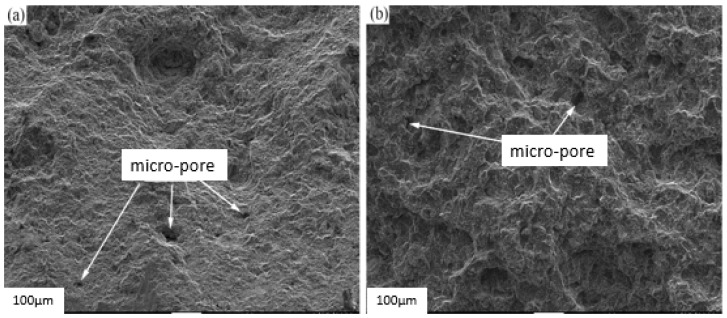
SEM image of the fracture surface of the specimen: the presence of micro-pores at the fracture, (**a**) one section of cracked specimen and (**b**) another section of cracked one.

The cross-sectional morphology of a photographed specimen is shown in Figure 12. The whole section was carefully observed, and no granular oxides were found on the section, but there were more tiny voids at the fracture. It is believed that the weakening of the mechanical properties of the specimen has a certain relationship with the micro-void.

The mechanical properties of aluminum alloys are affected by the porosity of castings. The experimental results indicate that the mechanical properties of die-cast aluminum alloys are related to porosity. As porosity increases, elongation decreases. The alloy’s elongation varies depending on the maximum pore size. This suggests that porosity significantly affects the alloy’s mechanical properties. The elongation increase in die-cast alloys is attributed to grain refinement because grain refinement leads to a reduction in pore size and a homogeneous distribution of porosity.

## 4. Conclusions

To enhance the durability of casting fittings, we evaluate the impact resistance of transmission and transformation fittings and the stability of the aluminum alloy material for long-term failure using the STP concept of the material. Through testing, we make a reasonable choice of materials and forming processes to improve the material’s STP and optimize the structure of vacuum die-casting molds. This increases the deformation absorption energy of the fitting parts, improves the anti-impact load performance, and reduces the incidence of rupture accidents.

(1) The results of the process test indicate that the VDCP can significantly reduce the formation of porosity defects during the die-casting process, thereby improving the quality and reliability of the parts. By utilizing high-strength materials and advanced molding processes, the surface quality of the parts can be improved, leading to enhanced anti-fatigue mechanical properties and increased reliability of the entire transmission line project.
(2) The test results indicate that the A356 aluminum alloy vacuum die-casting spacer bar has an average tensile strength of 246.52 MPa and an average elongation of 10.18%, with elongation exceeding 7%. Its STP value of 2509.57 MPa% is much higher than the typical value of the ZL102 aluminum alloy, and it greatly improved the strength and toughness of the product.
(3) The utilization of water-based mold release agents that are more environmentally friendly can resolve the challenges of releasing A356 aluminum alloy die-casting molds, thereby meeting the requirements for mass production.

## Figures and Tables

**Table 1:** Composition of a conventional ZL102 cast aluminum alloy ingot (wt.%).

Si	Fe	Cu	Mn	Mg	Ti	Zn	Al
10.0~13.0	0~1.00	≤0.30	≤0.50	≤0.10	≤0.20	≤0.10	Bal

**Table 2:** Chemical composition of an A356.2 cast aluminum alloy ingot (wt.%) (ASTM executive standard).

Si	Fe	Cu	Mn	Mg	Ti	Zn	Al
6.5~7.5	0~0.12	≤0.10	≤0.05	0.30~0.45	≤0.20	≤0.05	Bal

**Table 3:** Mechanical properties of two different materials with the VDCP.

Material	Process	Tensile Strength (MPa)	Elongation (%)	STP (MPa%)
ZL102	VDCP	157.67	3.5	551.84
A356	VDCP	246.52	10.18	2509.57

## Data Availability

Data are contained within the article.
